# Comparison of salivary and plasma adiponectin and leptin in patients with metabolic syndrome

**DOI:** 10.1186/1758-5996-6-19

**Published:** 2014-02-14

**Authors:** Supanee Thanakun, Hisashi Watanabe, Sroisiri Thaweboon, Yuichi Izumi

**Affiliations:** 1 Department of Oral Medicine and Periodontology, Faculty of Dentistry, Mahidol University, 6 Yodhi Str., Rajthewee, Bangkok 10400, Thailand; 2 Dental Center, Golden Jubilee Medical Center, Mahidol University, Bangkok, Thailand; 3 Department of Periodontology, Graduate School of Medical and Dental Sciences, Tokyo Medical and Dental University, Tokyo, Japan; 4 Department of Oral Microbiology, Faculty of Dentistry, Mahidol University, Bangkok, Thailand; 5 Global Center of Excellence Program for Tooth and Bone Research, Tokyo Medical and Dental University, Bangkok, Japan

**Keywords:** Saliva, Plasma, Adiponectin, Leptin, Metabolic syndrome

## Abstract

**Background:**

The relationship of saliva with plasma protein levels makes saliva an attractive diagnostic tool. Plasma levels of adiponectin and leptin in healthy individuals or diabetes mellitus patients have been previously reported. Nevertheless, salivary levels of these adipocytokines in patients with metabolic syndrome (MS) have never been investigated. This study was aimed to determine adiponectin and leptin levels in saliva and plasma from patients with metabolic syndrome, and evaluate any correlation of these levels with MS.

**Methods:**

Forty-six healthy and 82 MS patients were enrolled. Demographic data and blood biochemistries were recorded. Saliva and plasma adiponectin and leptin levels were analyzed by enzyme-linked immunosorbent assay (ELISA).

**Results:**

Adiponectin and leptin were higher in plasma than in saliva (*p* < .001). Plasma adiponectin was decreased and plasma leptin increased in patients with MS (*p* < .001). Salivary adiponectin and salivary leptin were not different between healthy subjects and MS patients (*p* = .619 and *p* = .523). Correlation between salivary and plasma adiponectin showed significant association (*r* = .211, *p* = .018) while salivary and plasma leptin had no correlation (*r* = -.161, *p* = .069). Significant correlation was observed between the salivary adiponectin/salivary leptin ratio and plasma adiponectin (*r* = .371, *p* < .001), but not with any component of MS. Increased triglyceride and waist circumference were associated with risk of having a low level of plasma adiponectin (OR = 1.009; 95% CI 1.002–1.015 and OR = 1.125; 95% CI 1.029–1.230). For leptin, body mass index and high-density lipoprotein cholesterol (HDL-C) were associated with a high level of plasma leptin (OR = 1.621; 95% CI 1.212–2.168 and OR = .966; 95% CI .938–.996). The OR for MS as predicted by plasma adiponectin was .928 (95% CI .881-.977).

**Conclusions:**

This study showed that salivary adiponectin and leptin do not correlate with MS. Although correlation between salivary and plasma adiponectin was observed, no association with MS was observed. Only plasma adiponectin may be useful for the prediction of MS.

## Background

Metabolic syndrome (MS) is a disease that poses significant risk of developing diabetes mellitus (DM) and cardiovascular disease (CVD). It is indicated by the presence of three or more of the following components: abdominal obesity, raised fasting glucose, dyslipidemia (raised triglyceride (TG) with lowered high-density lipoprotein cholesterol (HDL-C)), and raised hypertension. Waist measurement for abdominal obesity varies between gender and among different populations [1]. The number of patients with MS is expanding worldwide. The prevalence in developed and developing countries is comparable, ranging from 15.2% to 43.7% [1-4].

Many of the components of MS are connected through physiological pathways. Adiponectin, an anti-atherogenic and anti-inflammatory adipocytokine involved in glucose and lipid metabolism, improves insulin sensitivity [5]. Adiponectin levels in plasma are inversely correlated with visceral adiposity. Lower levels of adiponectin were observed in patients with high blood pressure, hyperglycemia, low HDL-C, and hypertriglyceridemia, and in obese patients with MS [6]. Brooks et al. showed that a low level of circulating adiponectin may be used as a possible biomarker for MS [7]. Leptin, an anti-obesity adipocytokine, regulates body weight by modifying energy levels and increasing metabolic rate while decreasing food intake. Most overweight and obese patients show resistance to leptin at the receptor level, and therefore have higher leptin levels than non-overweight individuals [8]. Serum leptin levels in patients with MS are higher than those in healthy controls [9]. Adiponectin and leptin levels show an inverse correlation with each other [10].

The relationship between protein levels in saliva and plasma makes saliva an attractive diagnostic tool that could be used as an alternative to blood in tests measuring biomarkers [11]. Currently, assays are available to analyze various salivary parameters. Measurement of adiponectin and leptin in saliva is simple, non-invasive, and may be an acceptable alternative to plasma sampling. Previous studies have reported successful measurement of adiponectin and leptin in saliva from healthy individuals or DM patients [12-15]. To the best of our knowledge, no previous studies have analyzed the levels of these biomarkers in saliva from patients with MS. Thus, the aim of this study was to determine adiponectin and leptin profiles from saliva and plasma in patients with MS compared with those of healthy patients. We aimed to measure correlations between salivary and plasma adipocytokines in both groups to determine if an association exists between these biomarkers and MS.

## Materials and methods

### Study participants

Patients being seen at the Golden Jubilee Medical Center (Mahidol University) were randomly assessed for MS. All participants were fully informed before completing their written consent document. The protocol and consent forms were approved by The Ethics Committee of Mahidol University and Tokyo Medical and Dental University, which conforms to the Helsinki Declaration (Reference Number: MU-DT/PY-IRB2011/134.3006, TMDU-IRB 2012/1108.860). Waist circumference was measured, and levels of TG, HDL-C, fasting plasma glucose (FPG), and blood pressure (BP) were evaluated from patient medical chart records. MS was diagnosed when three of the following five factors were present: (1) elevated waist circumference (≥85 cm in Thai men and ≥80 cm in Thai women); (2) elevated TG level (≥150 mg/dL); (3) reduced HDL-C (<40 mg/dL in men, and <50 mg/dL in women); (4) elevated blood pressure (systolic BP (SBP) ≥130 or diastolic BP ≥85 mmHg); and (5) elevated FPG (≥100 mg/dL) [16].

Eighty-two patients newly diagnosed with MS and never receiving medications were included in this study. Patients with systemic diseases as well as those who had received medications were excluded. Forty-six subjects with healthy status according to case selection procedure were recruited as a control group. Demographic data were collected. The height and body weight of participants were also recorded and body mass index (BMI) was calculated.

### Plasma collection

On the same date that peripheral vein blood was drawn to determine blood chemistries, a second blood sample was centrifuged to obtain plasma and a 1-mL aliquot was kept frozen (-80°C) for further adipocytokine analyses.

### Saliva collection

Unstimulated whole saliva was collected from all patients. Each individual was requested to abstain from eating, drinking, smoking, and brushing his/her teeth for at least 60 min prior to collection. Saliva samples were collected between 9 a.m. and 12 noon. Unstimulated whole saliva was collected using the drooling technique. Each subject rinsed their mouth with water before saliva collection, then the patient was asked to swallow to remove saliva from the mouth. The patient was seated upright, and leaned their head forward over a test tube with a funnel, allowing their saliva to drain into the tube. Whole saliva (~5 mL) was obtained from each individual. During saliva collection, the test tube was placed on ice. At the end of the collection, any remaining saliva in the patient’s mouth was expelled into the test tube.

### Saliva processing

Saliva obtained was centrifuged at 15,000 × g (MPW-65R, MPW.Med Instrument, Warszawa, Poland) for 15 min at 4°C to remove insoluble material. Supernatant was divided into 1-mL aliquots in pre-chilled cryotubes. The specimens were immediately frozen (-80°C) until analysis.

### Assay for adiponectin and leptin

Saliva and plasma were thawed at room temperature for adipocytokine measurement by enzyme-linked immunosorbent assay (ELISA). A commercial ELISA kit for human adiponectin and leptin (RayBio®, Norcross, GA) was used and the assay was conducted according to the manufacturer’s instructions with minor modification as previously reported [15]. Each sample of saliva and plasma from the same patient was run in the same experimental set and analyzed in duplicate. Plasma samples required 20,000-fold dilution for adiponectin and 100-fold dilution for leptin; saliva samples were analyzed without dilution.

### Data analysis

SPSS 16.0 software for Windows (SPSS Inc., Chicago, IL) was used for statistical analyses. Descriptive statistics were calculated and scatterplots created for each biomarker. All continuous variables were first assessed for normality using the Kolmogorov-Smirnov test and biomarker data were positively skewed as anticipated. Results are displayed as median with interquartile range. Comparison between saliva and plasma adipocytokine was performed using the Wilcoxon signed-rank test. Comparison of saliva or plasma adipocytokines and other variables between healthy patients and patients with MS was performed using the Mann–Whitney U test. Spearman’s Rho correlation coefficients and coefficients of partial correlation controlling for age, gender, and BMI were calculated to examine relationships between saliva and plasma adipocytokines and variables of MS. Logistic regression analysis controlling for the effect of covariates was further performed to ascertain the independent predictive value and impact of variables significantly correlated with plasma adiponectin levels, plasma leptin levels, and MS. A *p* value of < .05 was considered to be statistically significant.

## Results

### Characteristics of the study population

One hundred and twenty-eight patients were enrolled in this study. Eighty-two (64.06%) patients had MS. Of these, 43 patients (52.44%) had three of five components, 28 patients (34.15%) had four of five components, and 11 patients (13.41%) had all five components of MS. Patient characteristics and biomarker levels are summarized in Table 1. All blood chemistries had statistically significant differences between healthy subjects and patients with MS (Table 1).

**Table 1:** Characteristics of the study population and biomarker levels

	**Healthy subjects (n = 46)**			**Patients with metabolic syndrome (n = 82)**			***p*-value**
	**Total**	**Male**	**Female**	**Total**	**Male**	**Female**	
Gender	46	13	33	82	40	42	.024
Age (years)	44.50	52.00	44.00	48.00	49.00	48.00^‡^	.013
	(38.75-53.00)	(41.50-58.50)	(38.00-49.00)	(43.75-58.00)	(44.00-58.75)	(42.75-55.75)	
Body mass index (kg/m^2^)	21.82	22.00	21.81	27.26	26.97^#^	27.28^#^	<.001
	(20.22-22.89)	(20.97-23.55)	(19.42-22.81)	(24.56-29.53)	(24.68-29.20)	(23.96-30.10)	
Triglyceride (mg/dL)	73.00	72.00	73.00	181.50	205.50^#^	165.00^#^	<.001
	(57.75-89.00)	(64.00-95.00)	(56.50-86.50)	(136.00-290.50)	(141.50-304.00)	(128.00-244.00)	
High density lipoprotein cholesterol (mg/dL)	-	60.00	65.00	-	44.50^#^	44.50^#^	<.001
		(53.00-65.00)	(60.00-75.50)		(35.25-51.75)	(40.75-49.25)	
Low density lipoprotein cholesterol (mg/dL)	105.50	115.00	100.00	159.50	154.50^#^	162.50^#^	<.001
	(91.25-117.25)	(101.00-123.00)	(88.50-116.50)	(141.00-184.00)	(140.25-188.00)	(141.75-184.00)	
Total cholesterol (mg/dL)	182.00	191.00	178.00	233.00	232.00^#^	235.00^#^	<.001
	(169.75-193.25)	(177.50-195.00)	(165.50-191.50)	(210.25-260.00)	(212.25-259.75)	(207.75-260.25)	
Systolic blood pressure (mmHg)	117.00	118.00	116.00	134.00	135.00^#^	133.00^#^	<.001
	(109.00-125.25)	(109–126.50)	(109.00-125.00)	(119.75-141.25)	(120.50-141.75)	(117.75-141.25)	
Diastolic blood pressure (mmHg)	73.00	78.00	72.00	85.50	88.00^#^	81.50^#^	<.001
	(68.75-79.25)	(70.50-80.50)	(67.50-78.00)	(79.00-91.25)	(82.25-95.00)	(74.75-87.25)	
Fasting plasma glucose (mg/dL)	90.00	92.00	90.00	102.50	107.50^#^	100.00^#^	<.001
	(87.00-94.00)	(89.00-94.00)	(86.00-94.00)	(96.00-119.25)	(99.00-123.00)	(92.75-108.00)	
Waist circumference (cm)	-	81.00	75.00	-	91.00^#^	90.50^#^	<.001
		(73.50-82.00)	(69.50-78.50)		(86.00-95.75)	(86.75-94.50)	
Plasma adiponectin (μg/ml)	32.24	20.21	33.38	14.01	13.69^‡^	14.21^#^	<.001
	(17.96-46.84)	(9.842-41.33)	(21.13-48.64)	(8.09-14.010)	(5.38-20.79)	(9.66-28.07)	
Salivary adiponectin (μg/ml)	2.92	3.46	1.93	2.78	3.51	1.88	.619
	(7.91-6.23)	(1.74-7.03)	(5.62-4.38)	(1.05-6.48)	(1.49-9.09)	(8.17-6.25)	
Plasma leptin (ng/ml)	6.04	2.39	7.39	13.23	7.37^#^	21.70^#^	<.001
	(2.92-9.24)	(1.17-3.58)	(5.13-10.38)	(6.50-22.13)	(4.85-11.40)	(14.77-28.59)	
Salivary leptin (pg/ml)	33.80	38.51	31.21	34.88	43.17	29.59	.523
	(14.66-102.80)	(18.71-104.55)	(11.71-103.89)	(17.07-54.77)	(14.57-59.88)	(17.34-48.98)	

All data presented as median (25th-75th percentile).

^#^Significant difference from healthy same gender (p < .001).

^‡^Significant difference from healthy same gender.

### Adipocytokine profiles and correlation with MS components

A Kruskal–Wallis test revealed no statistically significant difference in the number of markers of MS across plasma adiponectin (ϰ^2^ = 2.857, df = 2, p = .240) or leptin (ϰ^2^ = 1.444, df = 2, p = .486), although plasma adiponectin levels were likely to be decreased when components of MS were increased. Consequently, all MS patients, despite exhibiting three, four, or five components of MS, were grouped together for data analysis.

**Figure 1:**
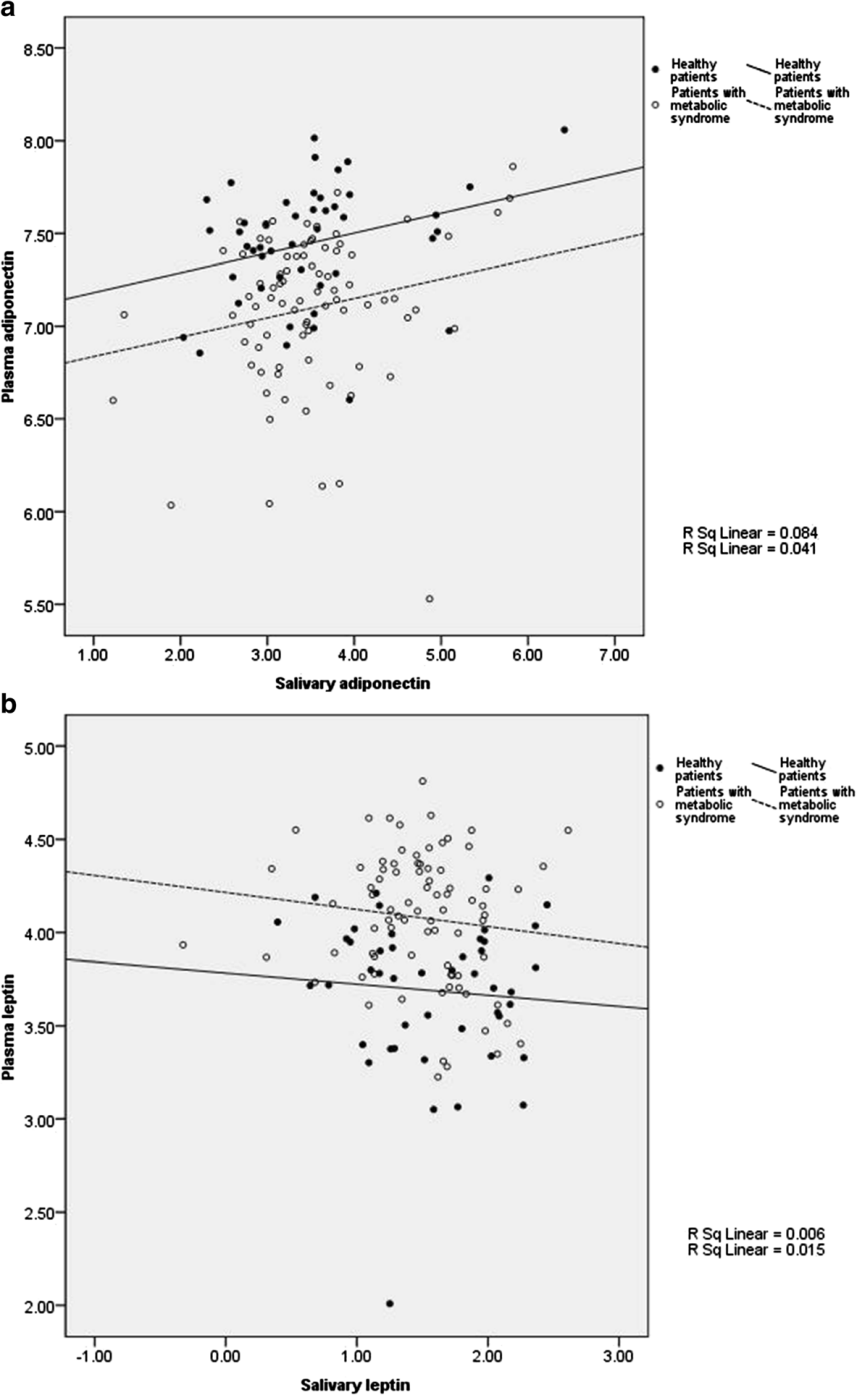
**Scatter plot demonstrating the association between salivary and plasma adiponectin levels (*r*** **= .211, *p*** **= .018) (a), salivary and plasma leptin levels (*r*** **= -.161, *p*** **= .069) (b), adjusting for age, gender, and BMI.** Values of salivary and plasma adiponectin and leptin have been log transformed.

Salivary and plasma levels of adiponectin and leptin are shown in Table 1. Adiponectin and leptin levels in plasma were significantly higher than those in saliva, both in healthy subjects and patients with MS (*p* < .001). Plasma adiponectin levels were decreased significantly in patients with MS compared with healthy individuals (*p* < .001) while salivary adiponectin levels were not significantly different (*p* = .619). In contrast to adiponectin, plasma leptin levels were significantly elevated in patients with MS compared with healthy individuals (*p* < .001). Salivary leptin levels showed no statistically significant difference compared with those of healthy persons (*p* = .523). Female patients in our study had higher plasma adiponectin and leptin levels compared with the male participants (*p* < .001, *p* = .004 respectively), while no significant difference of salivary adiponectin (*p* = .341) and leptin (*p* = .462) levels between male and female participants was observed. Spearman’s Rho correlation between salivary and plasma adiponectin levels showed significant association (*r* = .179, *p* = .043), and significance increased after controlling for age, gender, and BMI (*r* = .211, *p* = .018) (Figure 1a). Salivary and plasma leptin levels produced no significant correlation (*r* = -.161, *p* = .069) (Figure 1b), even after controlling for age, gender, and BMI (*r* = .000, *p* = .997).

When determining each component of MS, the correlations between plasma adiponectin and TG, HDL-C, diastolic blood pressure (DBP), and waist circumference were significant after adjusting for age, gender, and BMI, while salivary adiponectin levels did not correlate with age, gender, BMI, or any component of MS (Table 2). The relationship between plasma leptin, HDL-C, and waist circumference was also significant after adjusting for age, gender, and BMI, while salivary leptin levels did not correlate with any components (Table 2).

**Table 2:** Coefficients of bivariate and partial correlation between salivary and plasma adipocytokine values and various biomarkers of metabolic syndrome, controlling for age, gender (Model 1) and age, gender and BMI (Model 2)

	**Plasma adiponectin**			**Salivary adiponectin**			**Plasma leptin**			**Salivary leptin**		
	**Bivariate correlation**	**Partial correlation**		**Bivariate correlation**	**Partial correlation**		**Bivariate correlation**	**Partial correlation**		**Bivariate correlation**	**Partial correlation**	
		**Model 1**	**Model 2**		**Model 1**	**Model 2**		**Model 1**	**Model 2**		**Model 1**	**Model 2**
Age	NS	-	-	NS	-	-	NS	-	-	NS	-	-
Gender	r = .259 p = .004	-	-	-	-	-	r = .484 p < .001	-	-	-	-	-
Body mass index	r = -.315 p < .001	r = -.197 p = .027	-	NS	NS	-	r = .557 p = .000	r = .700 p < .001	-	NS	NS	-
Triglyceride	r = -.484 p < .001	r = -.290 p = .001	r = -.245 p = .006	NS	NS	NS	r = .334 p < .001	r = .316 p < .001	NS	NS	NS	NS
High density lipoprotein cholesterol	r = .476 p < .001	r = .269 p = .002	r = .242 p = .007	NS	NS	NS	r = -.308 p < .001	r = -.310 p < .001	r = -.263 p = .003	NS	NS	NS
Systolic blood pressure	NS	r = -.216 p = .015	NS	NS	NS	NS	NS	r = .251 p = .005	NS	NS	NS	NS
Diastolic blood pressure	r = -.333 p < .001	r = -.318 p < .001	r = -.267 p = .003	NS	NS	NS	NS	r = .282 p = .001	NS	NS	NS	NS
Fasting plasma glucose	r = -.352 p < .001	NS	NS	NS	NS	NS	r = .175 p = .048	NS	NS	NS	NS	NS
Waist circumference	r = -.332 p < .001	r = -.328 p < .001	r = -.291 p = .001	NS	NS	NS	r = .494 p < .001	r = .671 p < .001	r = .257 p = .004	NS	NS	NS

NS = Not Significant.

To comprehensively define other associations, the correlations of plasma adiponectin/plasma leptin, plasma adiponectin/salivary adiponectin, and salivary adiponectin/salivary leptin ratios were determined with BMI and each component of MS. Interestingly, plasma adiponectin/plasma leptin ratio were significantly correlated with all five components of MS (*p* < .05; data not shown in table). Looking specifically at salivary adipocytokines, the salivary adiponectin/salivary leptin ratio was also significantly associated with plasma adiponectin (*r* = .206, *p* = .019) and waist circumference (*r* = .251, *p* = .004). After controlling for age, gender, and BMI, a strongly significant correlation was still observed between the salivary adiponectin/salivary leptin ratio and plasma adiponectin (*r* = .371, *p* < .001).

### Association of adipocytokines with MS

To eliminate confounding effects, logistic regression analysis was performed. We divided the participants to create dichotomous groups based on low and high levels of plasma adiponectin and plasma leptin, using the median level of both adipocytokines as a cut-off point (18.488 μg/mL for adiponectin and 9.525 ng/mL for leptin). When low plasma adiponectin was used as a dependent variable, every unit increase of TG and waist circumference was significantly associated with risk of having low plasma adiponectin (odd ratio (OR) = 1.009; 95% confidence interval (CI) 1.002–1.015, and OR = 1.125; 95% CI 1.029–1.230, respectively) (Table 3). The relationship between leptin and components of MS was analyzed in the same manner using high plasma leptin as a dependent variable. BMI was associated with high plasma leptin by OR 1.621 (95% CI 1.212–2.168). Every unit increase in the HDL-C level was significantly associated with 3.4% decreased risk of having high plasma leptin (OR .966; 95% CI .938–.996). Additionally, high plasma leptin in female versus male patients persisted after the data was adjusted for other confounders (Table 4). Salivary adiponectin, salivary leptin, and the salivary adiponectin/salivary leptin ratio were not significant determinants for either plasma adipocytokine (Tables 3 and 4).

**Table 3:** Association of demographic variables, component of metabolic syndrome, and salivary adiponectin with low levels of plasma adiponectin in logistic regression model

			**Low level of plasma adiponectin (≤ 18.488 μg/ml)**			
**Independent variable**	**Plasma adiponectin (μg/ml) > 18.488**	**Plasma adiponectin (μg/ml) ≤ 18.488**	**Crude OR (95% CI)**	***p***	**Adjusted OR (95% CI)**	***p***
Age (years)	49.00 (43.25-58.00)	45.00 (39.00-53.00)	.967 (.932-1.003)	.075	.949 (.900-1.001)	.055
Gender						
Male (reference)	22 (17.19%)	31 (24.22%)				
Female	42 (32.81%)	33 (25.78%)	.558 (.274-1.136)	.108	.937 (.356-2.466)	.895
Body mass index (kg/m^2^)	23.45 (21.27-27.02)	25.30 (23.25-28.43)	1.124 (1.028-1.229)	.010*	.849 (.705-1.022)	.084
Triglyceride (mg/dL)	91.50 (62.00-154.50)	178.50 (104.50-293.00)	1.009 (1.005-1.014)	<.001*	1.009 (1.002-1.015)	.008*
High density lipoprotein cholesterol (mg/dL)	59.00 (47.25-66.00)	45.00 (37.25-55.75)	.969 (.946-.993)	.012*	1.006 (.978-1.035)	.667
Systolic blood pressure (mmHg)	127.00 (113.50-137.00)	126.50 (116.25-138.75)	1.007 (.984-1.030)	.565	.979 (.937-1.023	.344
Diastolic blood pressure (mmHg)	80.00 (72.00-85.75)	82.00 (75.00-88.75)	1.037 (1.000-1.075)	.051	1.002 (.933-1.075)	.962
Fasting plasma glucose (mg/dL)	94.00 (88.25-101.75)	99.50 (91.25-114.75)	1.008 (.999-1.017)	.082	1.003 (.993-1.013)	.538
Waist circumference (cm)	82.00 (74.25-91.00)	88.00 (82.25-94.00)	1.071 (1.029-1.114)	.001	1.125 (1.029-1.230)	.010*
Salivary adiponectin (μg/ml)	3.34 (1.05-6.49)	1.94 (.85-6.16)	1.000 (1.000-1.000)	.157	1.000 (1.000-1.000)	.231

Data presented as median (25th-75th percentile).

*statistical significance.

**Table 4:** Association of demographic variables, components of metabolic syndrome, and salivary leptin with high levels of plasma leptin in logistic regression model

			**High level of plasma leptin (> 9.525 ng/ml)**			
**Independent variable**	**Plasma leptin (ng/ml) ≤ 9.525**	**Plasma leptin (ng/ml) > 9.525**	**Crude OR (95% CI)**	***p***	**Adjusted OR (95% CI)**	***p***
Age (years)	46.50 (42.00-54.75)	48.00 (40.25-56.00)	1.010 (.975-1.047)	.581	1.043 (.971-1.120)	.246
Gender						
Male (reference)	38 (29.69%)	15 (11.72%)				
Female	26 (20.31%)	49 (38.28%)	4.774 (2.224-10.247)	<.001*	91.224 (13.397-621.151)	<.001*
Body mass index (kg/m^2^)	22.81 (21.18-24.74)	27.31 (23.87-29.96)	1.368 (1.207-1.549)	<.001*	1.621 (1.212-2.168)	.001*
Triglyceride (mg/dL)	89.00 (68.25-201.25)	160.50 (104.50-226.50)	1.002 (.999-1.005)	.124	.998 (.993-1.003)	.358
High density lipoprotein cholesterol (mg/dL)	60.00 (45.50-66.75)	47.50 (40.00-55.75)	.970 (.947-.994)	.015*	.966 (.938-.996)	.024*
Systolic blood pressure (mmHg)	125.50 (114.25-136.00)	128.00 (117.00-140.00)	1.012 (.989-1.036)	.310	.973 (.906-1.044)	.444
Diastolic blood pressure (mmHg)	80.00 (71.25-89.00)	81.00 (75.00-87.00)	1.007 (.973-1.043)	.684	1.037 (.942-1.142)	.458
Fasting plasma glucose (mg/dL)	94.00 (88.25-106.00)	99.50 (92.25-107.75)	.994 (.986-1.002)	.142	.987 (.969-1.005)	.143
Waist circumference (cm)	81.00 (74.00-86.00)	90.50 (86.00-94.75)	1.109 (1.060-1.161)	<.001*	1.099 (.978-1.236)	.113
Salivary Leptin (pg/ml)	45.26 (13.92-92.06)	30.19 (16.28-50.76)	.999 (.993-1.004)	.643	1.004 (.995-1.013)	.356

Data presented as median (25th-75th percentile).

*statistical significance

Finally, to assess the variables that could predict MS outcome, apart from standard criteria, logistic regression analysis was performed using several different models (Table 5). The fully adjusted OR for MS, as predicted by plasma adiponectin and controlling for age, gender, and BMI was .928 (95% CI, .881–.977; *p* < .05), indicating that there was a 7% decrease in the odds of having MS for each 1 μg/mL increase in plasma adiponectin. This model showed that age, BMI, and plasma adiponectin levels were significantly associated with a risk of MS, and could be used as the prognostic markers for MS by the equation: Log (odd) = -18.450 + .089(age) + .666(BMI) - .075(plasma adiponectin level).

**Table 5:** Risk of metabolic syndrome by age, gender, BMI, plasma, and salivary adipocytokines levels in logistic regression model

**Independent variable**	**Crude OR (95% CI)**	**Presence of metabolic syndrome**		
		**Adjusted OR (95% CI)**		
		**Model 1**	**Model 2**	**Model 3**
Age	1.047 (1.005-1.091)^**‡**^	1.071 (1.006-1.140)^**‡**^	1.115 (1.031-1.206)^#^	1.093 (1.008-1.186)^**‡**^
Gender (female)	.414 (.191-.897)^‡^	.903 (.278-2.933)	1.089 (.291-4.068)	.333 (.049-2.239)
Body mass index	2.504 (1.751-3.579)^##^	2.529 (1.725-3.708)^##^	2.393 (1.615-3.546)^##^	1.947 (1.280-2.961)^#^
Plasma adiponectin	.943 (.918-.969)^##^		.928 (.884-.974)^##^	.928 (.881-.977)^#^
Salivary adiponectin	1.000 (1.000-1.000)		1.000 (1.000-1.000)	1.000 (1.000-1.000)
Plasma leptin	1.179 (1.094-1.271)^##^			1.135 (.976-1.320)
Salivary leptin	.996 (.990-1.002)			.997 (.985-1.010)

^##^*p* < .001.

^#^*p* < .01.

^‡^*p* < .05.

## Discussion

Adiponectin and leptin were successfully detected in the saliva of patients with MS, although lower levels were observed compared with those in plasma. Salivary adiponectin, as well as the salivary adiponectin/salivary leptin ratio, was significantly correlated with its plasma levels, while salivary leptin levels were not. Between patients with MS and healthy individuals, significant differences were seen in adiponectin and leptin levels from plasma, but not from saliva. The levels of salivary adiponectin and leptin were in identical ranges to those found in previous studies that measured the levels in healthy subjects or in DM patients [13,14]. Our finding of a correlation between salivary and plasma adiponectin in this study is in agreement with previously published reports [13,17,18]. Toda et al. found that there was a significant correlation between plasma and salivary adiponectin levels in an older group of healthy patients [13]. Similar results have been recently reported in the studies of Mamali et al. and Akuailou et al., although the measured values of adiponectin were different, possibly due to saliva sample dilution and the characteristics of the patients [17,18].

However, uncorrelated salivary and plasma cytokines have been previously reported in other studies [19,20]. As in the present study, Aydin et al. saw that the levels of salivary leptin were almost the same as in plasma; no significant correlation between plasma leptin and salivary leptin was shown in all of the healthy patients in the study [21]. Nevertheless, others have seen a strong correlation between salivary and plasma leptin in healthy individuals, with this correlation significantly higher in plasma than saliva [12,22]. Dissimilar results in the correlation between salivary and plasma leptin levels might be explained by the laboratory method used (RIA vs ELISA), molecular structure of leptin, or the expression and secretion of leptin by the salivary glands themselves [12,22,23]. Furthermore, Randeva et al. reported circadian variation in the levels of salivary leptin. It reached low levels in the morning, the time at which saliva samples were collected in this study [12]. Thus, concentrations of salivary leptin do not always correlate well with plasma concentrations, as is seen with adiponectin. Autonomous production, structure modification, secretory pattern, or varying patient metabolism could reflect the discrepancies in the level and correlation of salivary and plasma leptin.

Regarding the correlation of salivary adiponectin and leptin with age or BMI, only two previous reports have discussed salivary adiponectin [13,17]. Similar to our study, others found salivary adiponectin was not significantly correlated with either age or BMI. Contrary to our study, salivary leptin correlation with BMI was described by a single previous study [12]. One interesting observation from our work was that the salivary adiponectin/salivary leptin ratio was significantly associated with waist circumference, although this association disappeared after controlling for age, gender, and BMI. No previous studies have documented this aspect, so it would be interesting for further studies to clarify this possible association of the salivary adiponectin/salivary leptin ratio with waist circumference. In addition, no previous reports directly investigated about salivary adiponectin and leptin with alcohol consumption, smoking habit or education level. However from our data analysis (data not shown), no statistically significant difference between healthy subjects and patients with metabolic syndrome according to these confounding factors and no correlation of these confounders with salivary levels of adipocytokines were found.

When we considered saliva and plasma adipocytokines between male and female patients, female patients in our study had higher plasma adiponectin and leptin levels compared with the male patients. Although slightly higher salivary adiponectin and leptin levels in men were observed in this study, no significant difference in salivary leptin and salivary adiponectin levels was observed between genders. Results showing that both saliva and plasma leptin levels are higher in female patients compared with male patients have been documented [12,21]. However, in the study by Randeva et al., the ratios of salivary and plasma leptin concentrations were higher in men [12]. Sex steroid hormones, body fat content or genetic differences might be responsible for this difference observed between men and women [12]. Despite this potential caveat, our findings extend the data of adiponectin and leptin levels in saliva of patients with MS.

Owing to the correlation of plasma adiponectin and leptin with MS components, this study found that plasma adiponectin and plasma leptin had distinct correlations with TG, HDL-C, DBP, and waist circumference. These results are consistent with many previous reports [24-30]. Others have shown there is a positive correlation of plasma adiponectin and HDL-C, and an inverse relationship between this adipocytokine with TG, waist circumference, and low-density lipoprotein-cholesterol (LDL-C) [31]. Similar to the data from Kawamoto et al. that showed an inverse association of TG and adiponectin [32], TG and waist circumference in our study were associated with the risk of having a low level of plasma adiponectin, while HDL-C was inversely associated with a high level of plasma leptin. Identical results were reported in a study by Wannamethee et al. where increased leptin levels were independently associated with a decrease in HDL-C [28]. Existing data for correlation between plasma adiponectin and age are controversial. Plasma adiponectin levels are reported as lower, unchanged, or higher as age increases [13,33,34], whereas plasma adiponectin levels were negatively correlated with BMI, and were higher in female patients than in male patients [25]. The present study noted a significant inverse association of plasma adiponectin levels with BMI, being higher in women than in men, while no association was observed with age. These results suggest that age, gender, and BMI could be the driving forces behind the correlations. Similar to previous studies, we reported the complex interaction of age, gender, and BMI with each component of MS [35-37]. The effect of sex hormones might explain this discrepancy, as it has been hypothesized that androgen inhibits adiponectin secretion [38].

Our results are in agreement with those of Schutte et al., who reported significant correlations of plasma adiponectin and plasma leptin with mean arterial pressure that disappeared after the effects of age and BMI were taken into account [37]. Moreover, our study showed that FPG was clearly not associated with plasma adiponectin and plasma leptin after adjustments for age, gender and BMI. While previous studies have shown that there are strong associations between low adiponectin levels and impaired FPG or insulin resistance, independent of age, gender, or BMI [24,29,33,34,39,40], meta-analysis revealed that higher adiponectin levels are associated with a lower risk of type 2 DM [41]. A controversial correlation between plasma leptin and BP and impaired fasting glucose or insulin resistance was published [27-29,40,42], however Wannamethee et al. found no association of plasma leptin and blood glucose, BP, and total cholesterol, which was identical to the results of this study and those of Wang et al. [28,29]. Controversies about correlations between adiponectin, leptin, and blood glucose might be due to different sample populations, and future studies are necessary to verify these results. Finally, the logistic regression model in this study showed that age, BMI, and plasma adiponectin, not salivary adiponectin, levels were significantly associated with the presence of MS. Similar to previous studies showing plasma adiponectin was a strong predictor of MS in both men and women [7,24,25,43].

The findings of the present study should be considered as preliminary, and subject to some limitations. First, this study was designed to be cross-sectional, which limits its ability to eliminate causal relationships between salivary and plasma adipocytokines and MS. Longitudinal studies are required to validate the diagnostic usefulness of saliva and plasma adiponectin and leptin for the detection of MS. Second, the sample size was not large; the patients who had four or five components of MS were fewer than those who had three components. This might reflect the insignificant difference of plasma adiponectin or leptin levels among each component group, a finding that is different from previous studies where plasma adiponectin levels decreased as components of MS increased [24,25], with leptin showing the opposite trend [33]. Our results need to be confirmed longitudinally and with a greater number of samples in order to find strong associations. Third, selection bias should be considered. The study group is not strictly a random population sample, being influenced by selection criteria and responses that might bias the results obtained from patients with MS versus healthy individuals. Last, another restriction is the lack of data for the homeostasis model assessment of insulin resistance (HOMA-IR). Owing to a laboratory limitation, adequate analysis for HOMA-IR has not been achieved.

## Conclusions

The present study suggests that measurement of adiponectin and leptin from saliva has uncertain accuracy as an alternative to plasma sampling for analytical fluid in patients with MS. Although correlation between salivary adiponectin and plasma adiponectin was presented, no association with MS components was observed with saliva samples. In particular, the reliability of salivary leptin as a biomarker was questionable. Clinical use of these salivary biomarkers currently has limitations, and needs further study before replacing plasma sampling. Measuring plasma adiponectin and leptin is still preferable. Plasma adiponectin may be a useful adjunct for the prediction of MS, as we observed a stronger association with MS than plasma leptin in this specific group of Thai patients with MS.

## Competing interests

The authors declare that they have no competing interests.

## Authors’ contributions

ST: designed and performed the experiments, analyzed the data and wrote the manuscript; HW and ST: participated in the design of the study; YI: conceived and designed the experiments, and helped to draft the manuscript. All authors read and approved the final manuscript.
